# Polyamine transporter in *Streptococcus pneumoniae* is essential for evading early innate immune responses in pneumococcal pneumonia

**DOI:** 10.1038/srep26964

**Published:** 2016-06-01

**Authors:** Aswathy N. Rai, Justin A. Thornton, John Stokes, Imran Sunesara, Edwin Swiatlo, Bindu Nanduri

**Affiliations:** 1 Department of Basic Sciences, College of Veterinary Medicine, Mississippi State University, Mississippi State, MS 39762, USA; 2 Department of Biological Sciences, Mississippi State University, Mississippi State, MS 39762, USA; 3 Center of Biostatistics and Bioinformatics, University of Mississippi Medical Center, Jackson, MS 39216, USA; 4 Division of Infectious Diseases, University of Mississippi Medical Center, Jackson, MS 39216, USA

## Abstract

*Streptococcus pneumoniae* is the most common bacterial etiology of pneumococcal pneumonia in adults worldwide. Genomic plasticity, antibiotic resistance and extreme capsular antigenic variation complicates the design of effective therapeutic strategies. Polyamines are ubiquitous small cationic molecules necessary for full expression of pneumococcal virulence. Polyamine transport system is an attractive therapeutic target as it is highly conserved across pneumococcal serotypes. In this study, we compared an isogenic deletion strain of *S. pneumoniae* TIGR4 in polyamine transport operon (*ΔpotABCD*) with the wild type in a mouse model of pneumococcal pneumonia. Our results show that the wild type persists in mouse lung 24 h post infection while the mutant strain is cleared by host defense mechanisms. We show that intact potABCD is required for survival in the host by providing resistance to neutrophil killing. Comparative proteomics analysis of murine lungs infected with wild type and *ΔpotABCD* pneumococci identified expression of proteins that could confer protection to wild type strain and help establish infection. We identified ERM complex, PGLYRP1, PTPRC/CD45 and POSTN as new players in the pathogenesis of pneumococcal pneumonia. Additionally, we found that deficiency of polyamine transport leads to up regulation of the polyamine synthesis genes *speE* and *cad in vitro*.

*Streptococcus pneumoniae* (pneumococcus) is the most common bacterial cause of community-acquired pneumonia (CAP), and otitis media worldwide^1^,^2^,^3^. In adults, pneumococcal pneumonia is the most common disease caused by pneumococcus with an estimated 900,000 cases reported annually in the United States, resulting in approximately 400,000 hospitalizations^4^,^5^,^6^. To date there are more than 90 pneumococcal serotypes with unique polysaccharide structure^7^. Currently available polysaccharide-conjugate vaccines (PCV) do not include all serotypes. Increased resistance to antibiotics such as penicillin, cephalosporin, and fluoroquinolones complicates treatment^8^,^9^. Serotype variability and genome plasticity enable pneumococci to adapt to host environments and immunological pressure, confounding intervention strategies. Therefore, protein based vaccine targets that are effective against all serotypes are attractive alternates to PCVs^10^. Although proteins such as pneumolysin, pneumococcal surface protein A (PspA), pneumococcal surface protein C (PspC), and histidine-triad proteins have been tested in animal models as potential pneumococcal vaccine candidates^11^,^12^,^13^, a licensed protein based vaccine for pneumococcal disease is still unavailable. Hence, there is a need to identify novel pneumococcal protein targets for vaccine development. Pathogen specific host proteins could also serve as targets for novel classes of antibacterial agents.

Polyamines are ubiquitous small cationic molecules that are important for growth and virulence of pneumococcus^14^,^15^. The most common intracellular polyamines, such as putrescine, spermidine and cadavarine, carry a net positive charge at physiological pH and form electrostatic bonds with negatively charged macromolecules, particularly nucleic acids, to maintain a stable conformation and regulate transcription among other processes^16^. In bacteria, polyamines are implicated in scavenging iron and free radicals, conferring acid resistance, promoting biofilm formation, helping escape from the phagolysosomes, and interactions with various components of cell envelopes^15^,^17^. Although the effect of polyamines on bacterial virulence has been studied in *Neisseria gonorrhoeae, Escherichia coli*, *Vibrio cholerae*, *Helicobacter pylori*, and *Shigella flexneri*^15^, the role of polyamines in pneumococcal pneumonia remains largely unexplored. Polyamine transport and synthesis genes are highly conserved among pneumococcal serotypes^18^ and are potentially attractive therapeutic targets. In pneumococcus, the polyamine transport operon *potABCD* consists of a membrane associated cytosolic ATPase (PotA), trans-membrane channel forming proteins (PotB and PotC) and the extracellular polyamine recognition domain (PotD)^15^. Deletion of *potD* in a type 3 pneumococcus resulted in severe attenuation of virulence in systemic and pulmonary models of pneumococcal disease^19^. In mice, PotD vaccination confers protection against colonization, pneumococcal pneumonia and sepsis^20^. PotD, is a potential next generation vaccine candidate and its efficacy increases in combination with several other pneumococcal protein candidates such as sortase A and glutamyl tRNA synthetase^13^,^19^,^20^,^21^. Recent studies showed delayed autolysis in a serotype 2 strain in which *potD* was deleted^22^. Although polyamines are implicated in a wide array of cellular processes, specific mechanistic roles for polyamines are yet to be assigned^17^.

It is known that pneumococci can invade lungs as early as a minute following intranasal challenge^23^. Our earlier findings showed that isogenic deletion of *potABCD* in TIGR4 (*ΔpotABCD*), a type 4 strain, led to attenuation in a mouse model of pneumococcal pneumonia 48 h following infection compared to the wild type (WT) strain, and active immunization with recombinant PotD affords protection against systemic infection^24^,^24^,^25^. The inability of polyamine transport mutants to survive in the host could be due to altered expression of pneumococcal genes regulated by polyamines, including the expression of virulence factors such as pneumolysin and capsular polysaccharides^18^. Differences in early host innate immune responses against the WT and polyamine transport deficient mutants could also explain the observed attenuation, but remain unknown.

In this study, we compared the early host immune responses to WT and polyamine transport deficient strain, in an intranasal challenge model of pneumococcal pneumonia in mice. Impaired polyamine transport in *S. pneumoniae* TIGR4 *∆potABCD* resulted in its clearance from the lung and blood by 24 h. Concentration of several cytokines/chemokines were higher at 4 h and 12 h in mice infected with *∆potABCD.* Consistent with this observation we found a significant increase in the infiltration of neutrophils in the lung. Comparative expression proteomics analysis of mouse lung tissue using 1D LC ESI MS/MS identified differential regulation of proteins involved in neutrophil killing and bacterial clearance such as PTPRC/CD45, PGLYRP1 and Ezrin-Radixin-Moesin, to name a few. In pneumococci, synthesis and transport mechanisms regulate intracellular polyamine concentrations^18^,^26^. We show upregulation of polyamine biosynthetic genes *in vitro*, in transport deficient *∆potABCD*. We conclude that polyamine transport in the pneumococcus is essential to evade early innate immune responses. A thorough understanding of dedicated polyamine transport in the pneumococcus is an attractive avenue for developing small molecule based intervention strategies that do not depend on host immune status.

## Results

### Clearance of a polyamine transporter mutant in a mouse model of pneumococcal pneumonia

**Figure 1:**
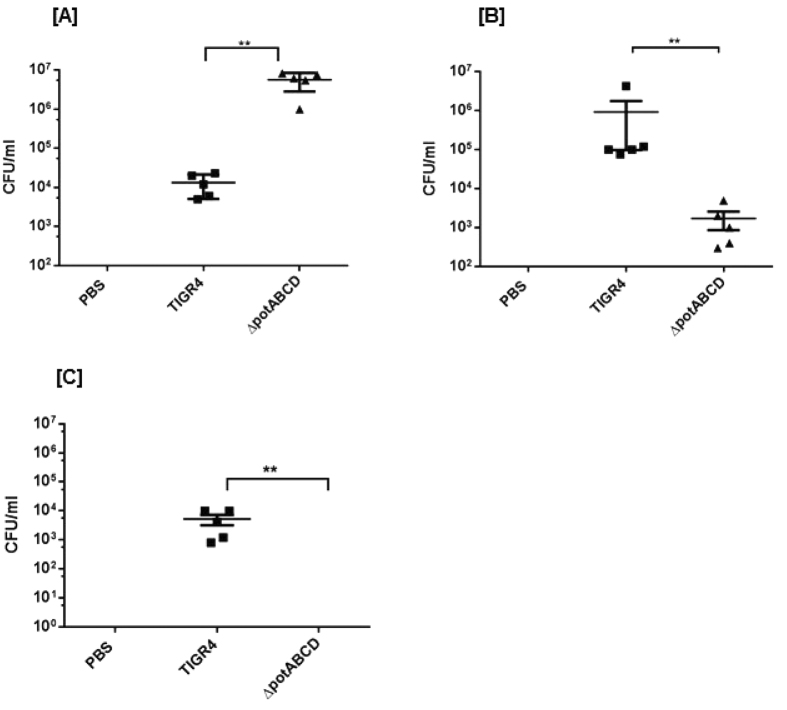
Transition of *S. pneumoniae* TIGR4 deficient in polyamine transport operon *(∆potABCD*) from nasopharynx to lungs. Recovery of *S. pneumoniae* TIGR4 or *∆potABCD* (CFU/ml) from lung homogenates from C57BL/6 mice (n = 5) infected i.n. with 10^7^ CFU bacteria at [**A**] 4 h, [**B**] 12 h and [**C**] 24 h. Data are represented as mean ± SEM. Each datum point represents results from one animal. Mann-Whitney test was used to calculate statisical significance (**p-value = 0.008).

*S. pneumoniae* is a commensal of the nasopharynx and invasive disease requires transition to sterile sites such as lungs and blood. To test if polyamine transport modulates pneumococcal transition and adaption to the lung environment, we infected mice with TIGR4 or polyamine transporter mutant intranasally to produce pneumonia. Lung and blood were aseptically collected in PBS at 4 h, 12 h and 24 h post infection (p.i.). We found significant differences in the bacterial burden at all time points in the lung of TIGR4 and ∆*potABCD* infected mice. Enumeration of bacteria from lungs showed a significantly higher number of ∆*potABCD* compared to TIGR4 at 4 h (Fig. 1A) post infection (p.i.). In contrast, TIGR4 ultimately persists better in the lower respiratory tract, as there were significantly more WT bacteria at 12 h (Fig. 1B) and 24 h p.i. (Fig. 1C) compared to ∆*potABCD*. The mutant strain was cleared by the host immune system by 24 h as demonstrated by the lack of bacteria recovered from lungs. Blood was sterile in all animals infected with WT and ∆*potABCD* at 4 h, 12 h and 24 h p.i. suggesting that neither WT nor ∆*potABCD* could invade the blood stream within 24 h of infection. Together, these results demonstrate that the deletion of the polyamine transporter in a capsular type 4 strain renders it more invasive yet susceptible to bacterial clearance mechanisms than the parent strain at early stages of infection.

### Polyamine transport in *S. pneumoniae* effects cytokine/chemokine expression in mice

Pneumococcal invasion of lungs results in complex early immune responses that can be characterized by cytokines and chemokines that induce pro- or anti- inflammatory responses necessary for bacterial clearance. The observed differences in bacteria in the lungs at 4 h and 12 h p.i. could result from differential cytokines/chemokine levels in the lung. To evaluate this, lung homogenates from animals challenged with TIGR4 and ∆*potABCD* were evaluated for 32 unique cytokines and chemokines (See methods). Consistent with the observed increase in the bacterial burden, the concentrations of G-CSF, LIF, IP-10, KC, GM-CSF, IL-5, IL-17 and MCP-1 were significantly higher in lungs from mice infected with *∆potABCD* at 4 h. p.i. (Table 1). Assessment of cytokine/chemokine levels at 12 h. p.i. showed elevated levels of IL-4, IFN-γ, MIG, GM-CSF, IL-17 and IL-7 relative to WT (Table 2). Majority of these cytokines and chemokines are associated with neutrophil recruitment response. Increased IL-17 levels correlate with the recruitment and activation of neutrophils and pneumococcal clearance in murine models of pneumococcal colonization^27^,^28^. Together, the 4 h and 12 h cytokine/chemokine data suggests a model in which ∆*potABCD* has decreased resistance to host immune response, potentially by decreased expression of virulence factors that facilitate evasion of early host cytokine responses and their effector functions.

### Differences in immune cell infiltration in mice infected with TIGR4 WT and ∆*potABCD* strains

**Figure 2:**
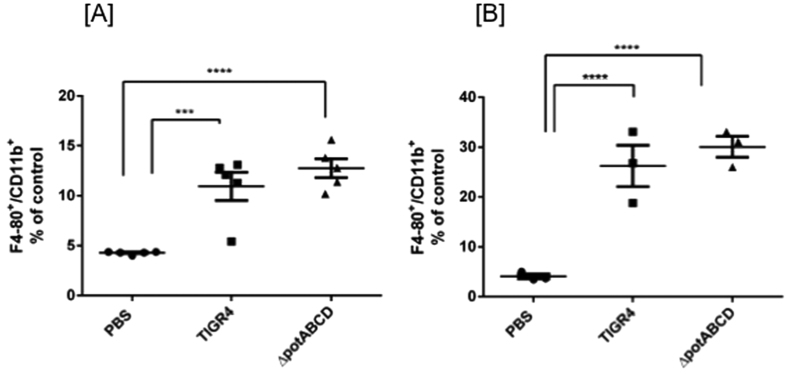
Evaluation of macrophage infiltration in lungs of C57BL/6 mice in response to infection with *S*. *pneumoniae* TIGR4 and *∆potABCD*. Expression of F4/80 and CD11b double positive cells from lung homogenates of C57BL/6 mice (n = 3) is shown. Data is expressed as percentage of F4/80, CD11b double positive cells compared to PBS at [**A**] 4 h and [**B**]12 h. Mice were challenged with 10^7^ *S*. *pneumoniae* TIGR4, *∆potABCD* or PBS. Each datum point represents results from one animal. Data are represented as mean ± SEM. One-way ANOVA and Tukey’s multiple testing comparison was used to calculate the statistical significance (***p-value = 0.0001; ****p-value = <0.0001).

**Figure 3:**
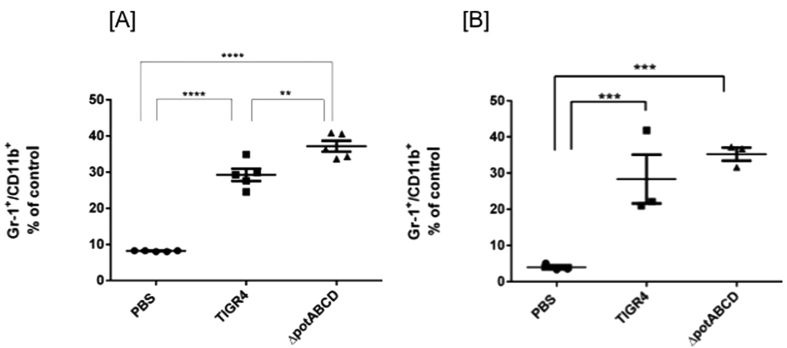
Evaluation of neutrophil infiltration in lungs of C57BL/6 mice in response to infection with *S*. *pneumoniae* TIGR4 and *∆potABCD*. Expression of Gr1 and CD11b double positive cells from lung homogenates of C57BL/6 mice (n = 3). Data is expressed as percentage of Gr1, CD11b double positive cells compared to PBS at [**A**] 4 h and [**B**]12 h. Mice were challenged with 10^7^ *S*. *pneumoniae* TIGR4, *∆potABCD* or PBS. Each datum point represents results from one animal. Data are represented as mean ± SEM. One-way ANOVA and Tukey’s multiple testing comparison was used to calculate the statistical significance (**p-value = 0.003; ***p-value = 0.0001; ****p-value = <0.0001).

We hypothesized that the comparatively higher bacterial burden at 4 h with ∆*potABCD* and the observed increase in chemokines and cytokines could lead to significant differences in the recruitment of neutrophils and macrophages to infected lungs. We tested this hypothesis using flow cytometry and determined changes in the distribution of neutrophils and macrophages in infected mice compared to control mice. Infiltrating non-alveolar macrophages in lung homogenates were quantified by counting cells expressing F4/80 and CD11b (Fig. 2) and neutrophils were quantitated by Gr-1 and CD11b expression (Fig. 3). We identified significantly higher number of neutrophils at 4 h p.i. with ∆*potABCD* mutant when compared to TIGR4 (Fig. 3A). No differences were observed at 12 h (Fig. 3B). Unlike neutrophil infiltration, there were no significant differences in the recruitment of infiltrating macrophages to the lung tissue with WT or ∆*potABCD* infection at 4 h or 12 h p.i. (Fig. 2A,B). Although we observed significant increase in the levels of MCP-1 in lung homogenates at 4 h (Table 1), a potential indication of macrophage and monocyte recruitment, previous studies have shown that these may not correlate with increased infiltration of macrophages^29^.

### Uptake of ∆*potABCD* by neutrophils does not require antibody opsonization

**Figure 4:**
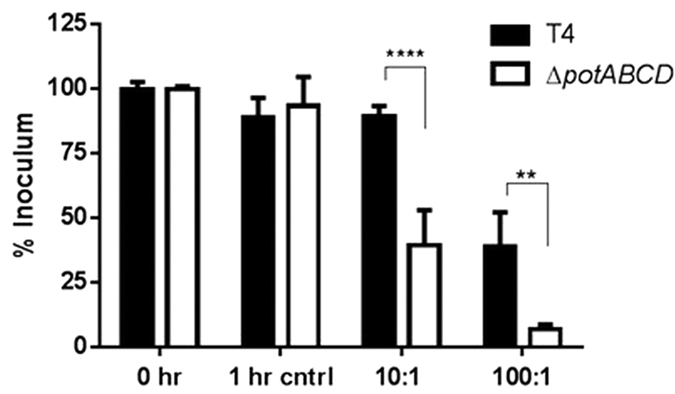
Opsonophagocytosis of *S. pneumoniae* TIGR4 and *ΔpotABCD* at two different bacteria: neutrophil ratios (1:10 and 1:100). 1 hr control represent the reaction mixture with neutrophils with no bacteria and T4 represents TIGR4. Data are represented as mean ± SEM. Two-way ANOVA and Sidak’s multiple comparison test were used to calculate the statistical significance (**p-value = 0.003; ****p-value = <0.0001).

Increased influx of neutrophils and clearance of ∆*potABCD* suggests a role for functioning polyamine transport in the WT for evading host neutrophil response. Increased uptake of ∆*potABCD* by neutrophils when compared to the WT could result in enhanced clearance of the polyamine transport deficient strain (Fig. 4). Our results show that there is ~50% increase in the uptake of ∆*potABCD* by murine neutrophils compared to WT. This uptake is dependent on phagocytosis and complement activation as addition of cytochalasin D and heat inactivation of serum had an inhibitory effect on bacterial uptake (See supplementary Fig. S1). However, we found that the uptake of ∆*potABCD* by neutrophils was independent of antibody opsonization as serotype 4 specific antibody was not required for phagocytosis (See supplementary Fig. S2B). Although WT bacterial numbers were reduced in the presence of neutrophils, opsonization with serotype 4 specific antibody was required for its efficient uptake (see supplementary Figs S1 and S2A). Hence, we conclude that polyamine transport is critical for protecting *S. pneumoniae* from opsonophagocytosis by the host.

### Polyamine transport is required for invasion of phagocytic and non-phagocytic cells

**Figure 5:**
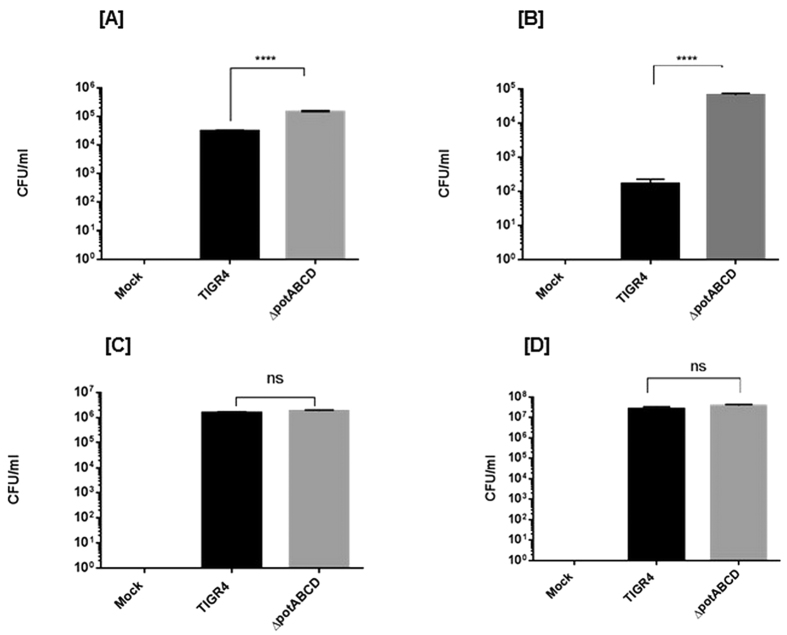
[**A**] Deletion of polyamine transporter enhances the uptake of *S*. *pneumoniae* TIGR4 by murine alveolar macrophage (AMJ2.C8) cells. Cells were cultured in appropriate medium to 90% confluency, and were infected with TIGR4 or *ΔpotABCD* at MOI of 1:10 for 2 h, followed by 1 h incubation with penicillin and gentamicin at 37 °C in 5% CO_2_. Cells were lysed in 0.0125% Triton X-100 for CFU enumeration. [**B**] Deletion of polyamine transporter enhances the invasion of *S*. *pneumoniae* TIGR4 in human BEAS.2B lung epithelial cells. Cells were cultured in appropriate medium to 90% confluency, and were infected with TIGR4 or *ΔpotABCD* at MOI of 1:10 for 2 h, followed by 1 h incubation with penicillin and gentamicin at 37 °C in 5% CO_2_. Cells were lysed in 0.0125% Triton X-100 for CFU enumeration. [**C**,**D**] Deletion of polyamine transporter does not affect the ability of *S*. *pneumoniae*. TIGR4 to adhere to murine AMJ2.C8 [**C**] and human BEAS.2B [**D**] lung epithelial cells. Cells were cultured as mentioned in 5A, infected with TIGR4 or *ΔpotABCD* at MOI of 1:10 for 2 h at 37 °C in 5% CO_2_, rinsed 3X in sterile PBS, and CFU enumerated. All assays were performed at least three times with two or more technical replicates. One-way ANOVA and Tukey’s multiple comparison tests were used to calculate the p-values. (****p-value = <0.0001, ns = p-value >0.05).

Our flow cytometry analysis of phagocytic cell infiltration was specific for non-alveolar macrophages. Unlike interstitial macrophages, alveolar macrophages express lower levels of CD11b rendering them CD11b low or negative for flow cytometric analysis^30^. Clearance of pneumococci from the lower respiratory tract also requires functional tissue resident alveolar macrophages^31^. Based on the observed differential uptake of WT and mutant by neutrophils, we asked if a similar difference could be observed in alveolar macrophages. Results from our invasion assays of mouse alveolar macrophages (AMJ2.C8 cells) at a MOI of 1:10 (cell: bacteria) show that *∆potABCD* is taken up more efficiently by macrophages (Fig. 5A) compared to WT.

Our *in vivo* analysis showed differences in the transition of WT and *∆potABCD* from nasopharynx to lungs. This could be the result of altered invasiveness of the mutant strain in epithelial cells. We tested this in BEAS.2B lung epithelial cells and found that *∆potABCD* could invade lung epithelial cells better than WT (Fig. 5B). However, we did not see any differences in the ability of TIGR4 or transport mutant to adhere to either murine alveolar macrophages or lung epithelial cells (Fig. 5C,D respectively). These results suggest the requirement for a functional polyamine transporter for uptake of TIGR4 by phagocytic alveolar macrophages and invasion of the lung epithelial cell barrier.

### Proteomic analyses of lung from mice infected with TIGR4 and *∆potABCD*

The propensity of pneumococci to establish infection in a healthy host relies, in part, on its inherent capacity to evade innate host defense mechanisms. Our results demonstrate that an intact polyamine transporter is critical for evading recruitment and uptake by host neutrophils. To identify specific changes in the lung proteome in response to TIGR4 and *∆potABCD* infection, we conducted mass spectrometry-based expression proteomics 4 h and 12 h p.i.

Proteomic analysis of lung tissue was performed on TIGR4 (n = 3), *∆potABCD* (n = 3) and sham/PBS treated (n = 3) for each challenge (4 h and 12 h). Compared to PBS treatment, a total of 44 and 82 proteins were identified as significantly altered (fold change ≥ 2-fold) in response to TIGR4 and *∆*potABCD, respectively, at 4 h p.i. (Tables 3, S1 and S3; p ≤ 0.05). Among these, 22 proteins were common to TIGR4 and *∆potABCD* infection and showed similar trend in expression (Table S3). Similarly, 92 and 66 differentially regulated proteins were identified 12 h p.i. (Tables 4, S2 and S4) with TIGR4 and *∆potABCD*, respectively, of which 46 proteins were shared between the two groups (Table S4). Several proteins were shared by WT and *∆potABCD* at both 4 h and 12 h such as ITGB2, S100A9, CORO1A, HP, CP, S100A8, CYBB, Chil3/Chil4, CFH, ELANE, Ngp, LTF and C3 (Tables S3 and S4). MMP9, required for phagocytosis of *S. pneumoniae* was also upregulated at comparable levels in WT and *∆potABCD* 4 h p.i.

Unique proteins down regulated in *∆potABCD* challenge at 4 h (>5-fold) included HNRNPH1, ACTN2, EZR, GPRC5A, POSTN, RPL30,TUBB4A, YWHAE, GSTM1, Macf1, NID2, PRELP, RDX, RTN3, SYPL1 and YWHAZ (Table 3). The expression of PTPRC/CD45 and PGLYRP1 were upregulated (>5-fold) in *∆potABCD* 4 h p.i. By 12 h, fewer proteins were identified with significant changes that were unique to ∆*potABCD* (Table 4). Upregulated proteins at 12 h included Igtp, Iigp1 and HLA-DQB1 while ITGA2 was down regulated (>5-fold).

The expression of APOE, LSP1, FETUB and VTN were upregulated (>5-fold) while the expression of EPB41L2, HBB, ABCD3 and FASN were down regulated (>5-fold) in WT TIGR4 by 4 h p.i. (Table S1). TIGR4 showed down regulation of Tmsb4x, NDUFA8, MPP1, UQCRHL, NCL, MTHFD1, Marcks, LRPAP1, ITGA8, CMKLR1, ATP2B1, AGK (>5-fold) and up regulation of C4A/C4B, ARPC4, NUCB1, C8B, TTN, C5, CKMT2, SERPINA1 by 12 h (Table S2).

**Figure 6:**
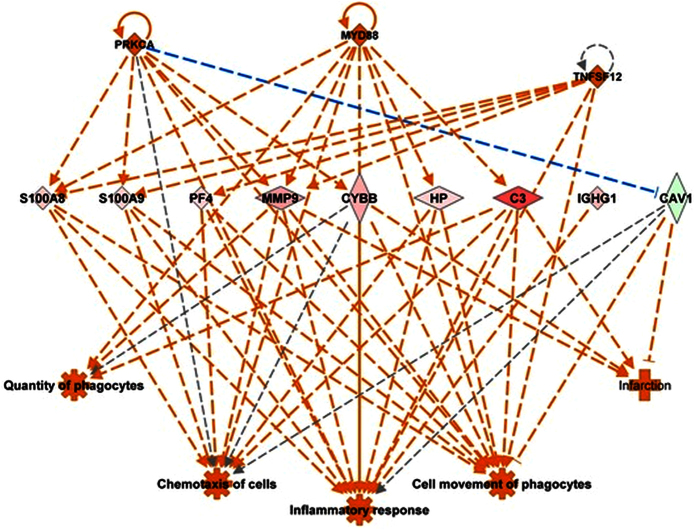
Predicted regulatory network effects identified from differential protein expression profile in lungs of C57BL/6 mice in response *S*. *pneumoniae ∆potABCD* 4 h p.i. Based on significant up- (shown in pink and red) down- (shown in green) regulation of lung proteins (represented by diamond shape), Ingenuity pathways analysis (IPA) predicted activation of upstream regulators such as MYD88, PRKCA and TNFSF12 (shown in orange). IPA’s regulator effects algorithm connected upstream regulators, proteins in our dataset to downstream functions to generate regulator effects hypotheses with a consistency score. The predicted top regulatory network (15.7 consistency score) in response to *∆potABCD* impacts inflammatory response, increase in quantity and movement of phagocytes which is consistent with the observed increase in neutrophils identified by FACS at 4 h p.i (Fig. 3).

**Figure 7:**
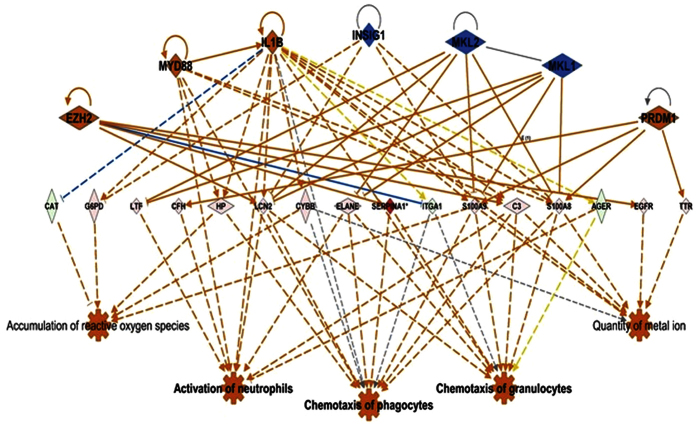
Predicted regulatory network effects identified from differential protein expression profile in lungs of C57BL/6 mice in response *S*. *pneumoniae* TIGR4 12 h p.i. Based on significant up- (shown in pink and red) down- (shown in green) regulation of lung proteins (represented by diamond shape), Ingenuity pathways analysis (IPA) predicted activation of upstream regulators such as EZH2, MYD88, IL-1β and PRDMI (shown in orange) and inhibition of INSIG1, MKL1 and 2 (shown in blue). IPA’s regulator effects algorithm connected the upstream regulators, proteins in our dataset to downstream functions to generate regulator effects hypotheses with a consistency score. The predicted top regulatory network (13.8 consistency score) in response to TIGR4 will impact chemotaxis of granulocytes, phagocytes and activation of neutrophils consistent with the establishment of infection, a delayed response when compared to *∆potABCD* 4 h (Fig. 6).

Differentially expressed mouse lung proteins in response to WT and *∆potABCD* strain were analyzed in the context of molecular function, signaling pathways and biological networks using Ingenuity Pathways analysis (IPA) software. Based on the expression pattern of proteins in a given dataset, IPA causal network analysis^32^ predicts either the activation or inhibition of upstream regulators. Causal network analysis with WT and *∆potABCD* at 4 h, predicted the activation of IL-6, TNF, MyD88 (activation Z-score ≥2.0). Predicted activation of interferon gamma was observed at 4 h and 12 h with *∆potABCD* and only at 12 h with WT. At 12 h IL-1β, TNF, MyD88 were predicted to be activated while MAPK12 was inhibited with WT. Based on the observed differences in recovered bacteria, neutrophil infiltration and cytokine concentrations at 4 h and 12 h with WT and *∆potABCD*, we expect to see a shift in host innate immune processes. In brief, a delay in early innate immune responses should be observed with WT. Regulator effects algorithm in IPA linked differentially expressed proteins, predicted activators/repressors to known impact/phenotype. Comparison of impact of observed differential expression on host immune responses with regulator effects algorithm identified that host response to *∆potABCD* at 4 h (Fig. 6) is comparable to the response to WT at 12 h (Fig. 7).

### *In vitro* analysis of *speE* and *cad* gene expression in *∆potABCD*

Polyamines regulate a wide array of cellular processes; consequently, their intracellular concentrations are tightly regulated^18^, by balancing extracellular transport with *de novo* synthesis of intracellular polyamines. Similar to *E. coli*, pneumococcus has SpeE that converts putrescine to spermidine, and Cad, which catalyzes the conversion of L-lysine to cadaverine. To address if lack of transport is compensated by synthesis, we evaluated the expression of *speE* and *cad* genes by qRT-PCR and found that compared to TIGR4, *speE* and *cad* levels were up regulated by 14.4 and 17.0 fold respectively in ∆*potABCD* (Table 5). Although these changes were found in cells grown *in vitro* we posit that cellular synthesis of polyamines by pneumococci growing in a host may wholly or partially compensate for diminished extracellular transport.

## Discussion

Despite intensive investigation into the natural history and pathogenesis of pneumococcal pneumonia, the ability of the pneumococcus to interact and adapt to the host milieu to establish infection is still confounding. Although much experimental focus has been placed on capsule, components of the cell wall, toxins, sensing systems and membrane transporters critical for adaption to host environments are just starting to gain attention in pneumococcal physiology and virulence^18^. Pneumococci can subvert innate immune responses by several mechanisms such as impeding the recruitment of phagocytic cells, circumventing intake by phagocytic cells, inducing the expression of host proteins that can block antimicrobial properties and inhibit/reduce complement activation and antibody-mediated opsonization^33^. Here we show that significantly reducing polyamine transport by deletion of a polyamine membrane transporter dampens several of these resistance mechanisms. In the present study, we used a polyamine transporter-deficient mutant to identify host innate immune effectors that normally serve to protect against pneumococcal pneumonia.

Our previous findings indicated that deletion of polyamine transport led to attenuation at 48 h^18^, in an intranasal challenge model of pneumococcal pneumonia in mice. In this study, we studied the kinetics of bacterial clearance in the lung in an intranasal challenge model and identified a significant difference in the bacterial load between the WT and *ΔpotABCD* as early as 4 h p.i. While the WT can persist in the lungs 24 h p.i., the mutant is cleared by host early innate immune responses. The larger number of mutant bacteria at 4 h reflects either a differential suppression of growth by the host or inherent differences in the two bacterial strains to replicate *in vivo*, early after entering the lower respiratory tract. Also, pneumococcal derived factors could produce a net growth advantage for the mutant bacteria early in the course of infection. The observed increase in the expression of specific cytokines/chemokines suggests that the host innate immune cells are differentially recruited to the sites of infection in pneumococci lacking potABCD. G-CSF coordinates the maturation and release of neutrophils from bone marrow^34^. Binding of KC to CXCR2 is required for releasing neutrophils into the circulation and G-CSF is known to facilitate this release by KC^35^. The up regulation of these neutrophil attractants suggest increased recruitment of neutrophils by polyamine transporter-deficient pneumococci. Although MCP-1 is shown to be involved in the recruitment of neutrophils and bacterial clearance in Gram- negative bacterial infections, studies on serotype 3 strains showed that MCP-1 is not required for recruitment of innate immune cells in pneumococcal pneumonia^36^. At 4 h and 12 h p.i. with *∆potABCD* we observed increased IL-17. Levels of IL-17 correlate with the recruitment and activation of neutrophils and pneumococcal clearance in murine models of pneumococcal colonization^27^,^28^. This finding suggests that intracellular polyamine levels in pneumococci are critical to bacterial defenses against host innate immune responses. Other physiological pathways in the pneumococcus will undoubtedly be shown to have similar roles in bacterial adaption and response to various host environments.

The clearance of bacteria in pneumococcal pneumonia is primarily mediated by neutrophils^37^,^38^. Increased early infiltration of neutrophils into the lungs and clearance of bacteria seen in transporter mutant infected mice suggested neutrophil killing as a potential mechanism of bacterial clearance. *S. pneumoniae* has evolved mechanisms to escape neutrophil extracellular traps (NETs) by degradation of the DNA scaffold through the endonuclease, endA^39^. Although we did not test neutrophil killing, our opsonophagocytosis assay clearly demonstrates that unlike WT, transporter-deficient mutants are taken up by neutrophils independent of opsonization. We show that transporter mutants are cleared by phagocytosis, as treating the neutrophils with cytochalsin D effectively inhibited the uptake of both WT and transporter mutant. Similarly, heat-inactivated serum reduced uptake of WT and transporter mutants by neutrophils, suggesting complement-mediated killing mechanisms. Our results demonstrate a role for ABC transporters such as PotABCD to alter the bacterial physiology to adapt to different host niches as infection is established. These adaptations can significantly alter the interface of pathogen and host immune responses. In lungs infected with WT pneumococci, we show increased expression of SerpinA1 (alpha-1-antitrypsin precursor), a known inhibitor of neutrophil serine proteases involved in bacterial killing (Table S2). This significant up regulation of a host resistance mechanism is lost with *ΔpotABCD*, which could in part explain its susceptibility to neutrophil killing. Further, increased expression of Leukotriene A4 hydrolase (Table 3) in *ΔpotABCD* infected animals could lead to increase in leukotriene mediated neutrophil ROS. This observation suggests that *ΔpotABCD* is possibly cleared by ROS mediated neutrophil killing mechanisms, which TIGR4 is resistant to^37^.

Proteome analysis of the lung showed significant up regulation of complement component C3 with both WT and mutant at 4 h, with a subsequent reduction at 12 h, which supports our neutrophil uptake data. C3 is critical for clearance of pneumococci^40^. Here we have shown that WT and polyamine transporter-deficient pneumococci both require complement for uptake by neutrophils, although the transport mutant succumbed quickly to phagocytosis (Fig. 4). This suggests that despite the differences in bacterial load, TIGR4 and *∆potABCD* elicit similar host responses. However, lack of polyamine transport could alter pneumococcal surface properties rendering it more susceptible to complement activation. This is further substantiated by the fact that the expression of ELANE, a neutrophil elastase, was significantly elevated in both WT and transport mutant at 4 h. Neutrophil elastase is a serine protease required for neutrophil killing^37^. Similarly, MMP9 is crucial for phagocytosis of *S. pneumoniae* by murine PMNS and intracellular bacterial killing^41^. The expression of MMP9 was comparable in WT and the mutant at 4 h, despite the differences observed in the early immune responses.

The proteome of mouse lungs challenged with *∆potABCD* parallels observations with immunoassays. Our proteomic screen revealed unique proteins that were differentially regulated in *∆potABCD* at 4 h. We found up regulation of PTPRC/CD45 in *∆potABCD* relative to WT at 4 h (Table 3). PTPRC/CD45 participates in the clearance of *Staphylococcus aureus* in animal models^42^. *Ptprc*^*−/−*^ mice display impaired neutrophil recruitment, weaker host defense and die prematurely^43^. The observed increase in the levels of PTPRC/CD45 may correlate with increased recruitment of neutrophils and clearance of *∆potABCD* by 24 h. Similarly, the expression of PGLYRP1 was up- regulated in *∆potABCD* relative to WT at 4 h. PGLYRP1 is an antimicrobial innate immunity protein commonly implicated in allergic asthma. Interestingly, *pglyrp1*^*−/−*^ mice are compromised in the recruitment of neutrophils, lymphocytes, eosinophils and macrophages to lung^44^, thereby substantiating increased recruitment of neutrophils by *∆potABCD* at 4 h p.i. Though not detected above baseline in TIGR4 after 4 h, we observed elevated expression of PGLYRP1at 12 h.

The majority of differentially regulated proteins unique to mice challenged with *∆potABCD* were down regulated at 4 h (Table 3). For example, periostin (POSTN) is a secreted extracellular matrix protein commonly associated with bronchial hyperresponsiveness and subepithelial fibrosis in asthmatic patients^45^,^46^ and its concentration is thought to correlate with disease severity^45^. *Postn*^*−/−*^ mice exhibit reduced inflammation of the airways in response to allergens such as house dust mites^45^. The observation that POSTN expression is significantly reduced in animals challenged with *∆potABCD* suggests its requirement in establishing infection by WT TIGR4 in lungs. We found all three components of the Ezrin-Radixin-Moesin (ERM) complex significantly down regulated in *∆potABCD* challenged animals 4 h p.i. Ezrin connects apical membranes to the cytoskeleton by crosslinking to actin filaments. *Listeria monocytogenes* effectively use the ERM complex to link the actin in their tail to membrane cytoskeleton enabling cell-cell spread^47^. Impairing the ERM complex results in inefficient establishment of infection^47^. Consequently, *Listeria monocytogenes* requires the ERM complex to evade host immune responses. Ezrin and radixin were significantly down regulated by 10- and 5-fold respectively (Table 3) and moesin levels down significantly by 1.7-fold (data not included, as it did not meet the ≥2-fold cut off). *Neisseria meningitidis* recruit ERM proteins that inhibit the formation of the endothelial docking structures critical for leukocyte diapedesis. When these docking sites with endothelial cells were eliminated by a dominant negative approach, leukocyte diapedesis was inhibited^48^. Taken together, the down regulation of Ezrin, Radixin and Moesin with *∆potABCD* suggest a novel mechanism by which pneumococci can escape host innate immunity and spread infection in the lung by regulating the expression of ERM complex. Although ERM complex seems to be critical for TIGR4 to establish infection, the pathogen-directed differential expression of this and other host factors warrants further investigation.

Previous studies showed that *ΔspeE* and *Δcad* were severely attenuated in a manner similar to Δ*potABCD*, in a mouse model of pneumococcal pneumonia^18^. Polyamine levels are stringently controlled by intracellular synthesis and transport mechanisms in pneumococci^18^,^26^. We found increased expression of *speE* and *cad* genes in *ΔpotABCD in vitro.* We previously reported proteomics of *ΔpotABCD*, and showed the increased expression of capsular polysaccharide biosynthesis proteins, pneumolysin and pneumococcal surface protein A^18^. The contribution of these proteins to the observed differences in the host innate immune response remains open for investigation.

In summary, deficiencies in polyamine transport cause attenuation of virulence in murine models of pneumonia^18^, and genes involved in polyamine transport appear to be conserved within the species, providing a potential new class of broad-based vaccine candidates or therapeutic targets. However, to leverage this knowledge for vaccine development, a better understanding of how extracellular polyamine transport promotes invasive infection is necessary. This will require an understanding of polyamine dependent expression of pneumococcal genes, and host immune mechanisms in pneumonia. This study focused on host response during infection and identified reduced resistance to neutrophil killing in polyamine transport deficient pneumococci. Comprehensive description of host-pneumococcal interactions responsive to altered polyamine metabolism will be critical for therapies that reduce the global disease burden in public health domain due to this important human pathogen.

## Materials and Methods

### Bacterial strains and growth conditions

*Streptococcus pneumoniae* serotype 4 strain TIGR4 was used in this study^49^. All strains were grown in Todd-Hewitt broth supplemented with 0.5% yeast extract (THY) or on 5% sheep blood agar (BA) plates. An isogenic mutant of TIGR4 deficient in the polyamine transport operon *potABCD* was generated by PCR-ligation mutagenesis as described previously^50^. Briefly, PCR primers were designed to amplify chromosomal DNA 600 nt 5′ to the start codon and 600 nt 3′ from the transcription termination site of *potABCD*. Erythromycin resistance gene (*ermB*) was amplified from pJY4163^51^. Genomic pieces were joined by gene splicing by overlap extension (SOEing) PCR^52^ using a forward primer of the upstream flanking region and the reverse primer of the downstream flanking region. The recombinant product was transformed into TIGR4 as described previously^53^. Transformants were selected on BA plates with erythromycin (0.5 μg/ml) by an overnight incubation at 37 °C with 5% CO_2_. The identity of mutant bacterial colonies were confirmed by PCR and sequencing. There was no significant difference between the WT and *∆potABCD* growth in THY as reported previously^18^.

### Mouse model of pneumonia and enumeration of CFU/ml

C57BL/6mice were used in all studies (8–10 week old). All animal care and experiments were performed in accordance with the protocols approved by Mississippi State University’s Institutional Animal Care and Use Committee (IACUC # 13–064). Briefly, mice were anesthetized with isoflurane and 50 μl of phosphate buffered saline (PBS) containing 10^7^ CFU of bacteria were inoculated in the nares. Mice were sacrificed at 4 h, 12 h and 24 h p.i. by CO_2_ asphyxiation. Lungs were removed, rinsed in PBS and homogenized in Hanks Balance Salt Solution (HBSS). Lung homogenates and blood were serially diluted and plated on BA plates to enumerate CFU/ml. Blood samples were plated on BA to ensure the absence of bacteria in blood. The results were validated by at least two independent experiments with a group size of 5 animals.

### Flow cytometry

Lung homogenates were prepared in Hank’s balanced salt solution (HBSS) with 0.2% BSA (low endotoxin and FFA free) using a handheld homogenizer (Cole Palmer, Vernon Hills, IL). Cells were passed through a 100 μM nylon filter, washed in RPMI media and resuspended in 0.5 ml of ACK lysis buffer (Life Technologies, Carlsbad, CA). The lysate was rinsed in PBS, filtered through a 35 μM filter and stained for 30 min at 4 °C with PE conjugated antibodies against CD11b (BDBiosciences, San Jose, CA), APC conjugated CD45, FITC conjugated Ly6G or PE-Cy7 conjugated F4/80 (Miltenyi Biotec, San Diego, CA). Viability was determined by a LIVE/DEAD dye, e-flour 780 (Affymetrix Viability was determined by a LIVE/DEAD dye, e-flour 780 (Affymetrix, Santa Clara, CA). Fluorescent minus one controls were used to set gating boundaries. Isotype matched control beads were used to account for nonspecific staining and 50,000 events were analyzed. Analysis was performed with FACSAria III flow cytometer (BD Biosciences, San Jose, CA) and data analyzed by FlowJo software (Tree Star, Inc., Ashland, OR). The results were validated by two independent experiments with a group size of at least 3 animals per challenge.

### Cytokine profiling

Cytokine profiling of samples were performed using MILLIPLEX MAP mouse cytokine/chemokine magnetic bead panels (MCYTMAG-70K-PX32) based on the Luminex xMAP technology (Millipore Corp., Billierica, MA) using standards and controls for each cytokine and chemokine provided by the manufacturer. The cytokine panel includes VEGF, Eotaxin, G-CSF, GM-CSF, IFN-γ, IL-1α, IL-1β, IL-2, IL-3, IL-4, IL-5, IL-6, IL-7, IL-9, IL-10, IL-12 (p40), IL-12 (p70), IL-13, IL-15, IL-17, IP-10, KC-like, LIF, LIX, MCP-1, M-CSF, MIG, MIP-1α, MIP-1β, MIP-2, RANTES and TNF-α. Concentrations are reported in pg/ml. Assays were performed with lung homogenates as per manufacturer’s instructions using at least five biological and two technical replicates. Data acquisition was done using xPonent and Milliplex analyst (Millipore Corp., Billierica, MA ). Sample wells with less than 20 pg/ml were invalidated. Exploratory data analyses enabled logarithmic base 2 transformations for all continuous variables. Linear regression analysis was used to test the comparisons between TIGR4 and *ΔpotABCD.* Sensitivity analysis was done using non-parametrics methods and found similar qualitative results. Stata 13.1 (Stata Corporation, College Station, TX) was used to conduct statistical analysis. The significance level was set at 0.05.

### Bacterial Adhesion and Invasion Assay

Bacterial adhesion and invasion were performed (n = 3) as described previously^54^ on BEAS.2B, human lung epithelial cells (ATCC-CRL-9609) and mouse alveolar macrophages AMJ2-C8 (ATCC-CRL-2455). Cells were cultured in 10% fetal calf serum (FCS) supplemented Dulbecco modified Eagle medium (DMEM) to 90% confluency in 12-well plates at 37 °C in 5% CO_2_. Cells were challenged with TIGR4 and *ΔpotABCD* at an MOI 1:10 (cell: bacteria) and incubated for 30 min for adherence or 2 h for invasion at 37 °C in 5% CO_2_ and washed with PBS. Invasion assay mixtures were further incubated for 1 h with gentamicin sulfate (200 μg/ml) then washed in PBS. Cells were lysed in 100 μl of 0.0125% Triton X-100 and the lysate was plated on BA for CFU enumeration.

### Proteomics

Lungs were harvested from infected and PBS/sham animals 4 h, 12 h p.i. Total proteins were isolated from uninfected, TIGR4, and ΔpotABCD infected groups (n = 3) by incubation in NP-40 lysis buffer (0.5% NP-40, 150 mM NaCl, 20 mM CaCl2·2H_2_O, 50 mM Tris, pH 7.4) for 20 min on ice. Protein concentration from the supernatant was determined and 100 μg was precipitated with methanol and chloroform. Protein samples were solubilized in 8M urea, reduced and alkylated, and were trypsin digested. Tryptic peptides were desalted using a C8 peptide macrotrap (Michrom BioResources Inc., Auburn, CA), eluted in 0.1% triflouroacetic acid, 95% acetonitrile, vacuum dried and resuspended in 0.1% formic acid. Tryptic peptides were subjected to 1D nano-LC ESI MS/MS analysis using an iontrap LTQ mass spectrometer (see supplementary methods). Liquid chromatographic (LC) analysis to separate peptides was performed using C18 column (Thermo Scientific, Waltham, MA ) controlled by Proxeon Easy n-LC (Thermo Scientific, Waltham, MA ). Peptides were eluted using a 180 min gradient at with 99.9% acetonitrile, 0.1% formic acid at a flow rate of 400 nl/min and introduced into an LTQ-OrbiTrap Velos mass spectrometer (Thermo Scientific, Waltham, MA ). Full scan MS spectra (60,000 resolution and m/Z 350–1,600 amu) were analyzed by Orbitrap, and the ions were selected for collision-induced fragmentation (CID) in the LTQ at normalized collision energy of 35% and activation time of 30 msec. Tandem mass spectra were extracted, charge state deconvoluted and deisotoped by Thermo Proteome Discoverer 1.3 (Thermo Scientific, Waltham, MA ). Sequest was set up to match mass spectra and tandem mass spectra (see supplementary information) against a non-redundant mouse protein database appended with *S. pneumoniae* proteins and common laboratory contaminants. A reversed decoy database was utilized to assess false discovery rate for peptide identification. Scaffold 4 (Proteome Software, Portland, OR) was used to filter Discoverer output. Peptide identifications were accepted if they could be established at greater than 92.0% probability to achieve an FDR less than 0.5% by the Scaffold Local FDR algorithm (see supplementary tables S5B–8B). Protein identifications were accepted if they could be established at greater than 99.0% probability and contained at least 2 identified peptides (see supplementary tables S5A–8A). Significant changes in protein expression between uninfected control versus TIGR4 and uninfected control versus *ΔpotABCD* at 4 h and 12 h were identified by Fisher’s exact test at a p-value of ≤0.05 and fold change in protein expression was calculated using weighted normalized spectra with 0.5 imputation value. The PRoteomics IDEntifications (PRIDE) database is a centralized, standards compliant, public data repository for proteomics data. The mass spectrometry proteomics data have been deposited to the ProteomeXchange Consortium^55^ via the PRIDE partner repository with the dataset identifier PXD002300 and 10.6019/PXD002300. Significantly altered lung proteins were analyzed using Ingenuity Pathways Analysis (IPA) as was done earlier^56^ (see supplementary methods). Based on the distinct up- and down regulation pattern of protein expression, IPA predicted activation of upstream and downstream regulators and utilized the regulator effects algorithm to connect identified upstream regulators with proteins in a dataset and downstream functions to generate a hypothesis with a consistency score. The predicted regulatory networks helped interpret the impact of observed protein expression changes on host response.

### Isolation of murine neutrophils

Murine neutrophils were isolated as previously described^37^. In brief, mice were intraperitoneally injected with 1 ml of 10% casein in PBS. A second dose was administered after 24 h and neutrophils were harvested 2 h following the second dose. Murine polymorphonuclear leukocytes (PMNs) were collected by lavage of the peritoneal cavity with Hanks buffer (no Ca^++^ and Mg^++^) supplemented with 0.1% gelatin. Neutrophils were enriched by Ficoll density gradient, washed with 5 ml of Hanks buffer (no Ca^++^ and Mg^++^) with 0.1% gelatin and resuspended in Hanks buffer (Ca^++^ and Mg^++^) and 0.1% gelatin.

### Opsonophagocytosis

Opsonophagocytic (OPH) killing assays were performed as described previously^37^. In brief, 10^3^ bacterial cells (in 10 μl) were preopsonized with Type 4 specific (Hyp4M3) monoclonal antibody^57^ unless mentioned otherwise (gift from Dr. Moon H. Nahm, The University of Alabama, at Birmingham) for 30 min at 37 °C. Neutrophils were then added at 1:10 and 1:100 (bacteria:neutrophils) and incubated for 1 h at 37 °C with rotation. Percent survival was determined relative to control without neutrophils. Cytochalasin D (20 μM) was used to pretreat neutrophils (30 min at 37 °C), and serum was heated at 56 °C for 30 min to inactivate complement.

### Quantitative Real Time PCR

The primers used for quantitative PCR (qRT-PCR) are listed in Table S9. In brief, total RNA was purified from mid-log phase cultures of TIGR4 and *ΔpotABCD* grown in THY media using the RNeasy Midi kit and QIAcube (QIAGEN, Valencia, CA). Purified total RNA (7.5 ng/reaction) was transcribed into cDNA and qRT-PCR was performed using the SuperScript™ III Platinum® SYBR® Green One-Step qRT-PCR Kit (Fisher Scientific, Pittsburgh, PA) according to manufacturer’s instructions. Relative quantification of gene expression were obtained using the Stratagene Mx3005P qPCR system (Agilent, Santa Clara, CA). Expression of target genes, *speE* (SP_0918) and *cad* (SP_0916) were normalized to the expression of *gdh* (glucose-6-phosphate 1-dehydrogenase, SP_1243) housekeeping gene and fold change determined by the comparative Ct method.

### Statistical Analysis

GraphPad Prism (version 5.02 for Windows, GraphPad Software, USA) was used for all statistical analysis performed unless mentioned otherwise. Statistical significance (p-values) were calculated as described in the figure legends.

## Additional Information

**How to cite this article**: Rai, A. N. *et al.* Polyamine transporter in *Streptococcus pneumoniae* is essential for evading early innate immune responses in pneumococcal pneumonia. *Sci. Rep.* **6**, 26964; doi: 10.1038/srep26964 (2016).

## Supplementary Material

Supplementary Information

Supplementary Table S5A and S5B

Supplementary Table S6A and S6B

Supplementary Table S7A and S7B

Supplementary Table S8A and S8B

## Figures and Tables

**Table 1:** Comparison of significantly altered cytokine/chemokine concentrations in lung of C57BL/6 mice infected with TIGR4 WT and *∆potABCD* strains 4 h p.i.

Cytokines/ Chemokines	TIGR4 Geometric Mean (SD) (pg/ml)	Δ*potABCD* Geometric Mean (SD) (pg/ml)	p-value
IP-10	190.01 (1.69)	1629.2 (2.92)	0.004
GM-CSF	97.68 (3.52)	1287.1 (1.98)	0.004
IL-5	34.78 (1.71)	127.11 (1.64)	0.004
KC	729.11 (2.32)	3420.5 (1.54)	0.006
LIF	35.75 (1.00)	256 (2.36)	0.01
G-CSF	652.57 (2.85)	3420.5 (1.5)	0.011
MCP-1	210.83 (1.55)	661.68 (2.01)	0.014
IL-17	11.48 (3.39)	69.56 (1.88)	0.019

Data shown represents the geometric means (SD) of concentration in pg/ml and represents values from 5 animals in each group with the significance level set at 0.05.

**Table 2:** Comparison of significantly altered cytokine/chemokine concentrations in lung of C57BL/6 mice infected with TIGR4 WT and *∆potABCD* strains12 h p.i.

Cytokine/ Chemokine	TIGR4 Geometric Mean (SD) (pg/ml)	Δ*potABCD* Geometric Mean (SD) (pg/ml)	p-values
IL-4	0.57 (1.44)	1.82 (1.5)	0.001
IFN-Υ	25.63 (2.77)	512 (3.72)	0.004
IL-17	7.51 (5.35)	86.82 (1.59)	0.014
MIG	891.44 (2.33)	2896.3 (213.92)	0.021
GM-CSF	106.15 (3.52)	544.95 (1.36)	0.023
IL-7	5.97 (1.63)	11.87 (1.46)	0.039

Data shown represents the geometric means (SD) of concentration in pg/ml and represents values from 5 animals in each group with the significance level set at 0.05.

**Table 3:** Significant changes in the lung proteome that are unique to *ΔpotABCD* 4 h p.i.

Protein	Description	Accession	Δ*potABCD/PBS* (Fold Change)
HNRNPH1	heterogeneous nuclear ribonucleoprotein H1 (H)	Q8C2Q7	−16.7
ACTN2	actinin, alpha 2	Q9JI91	−10
EZR	ezrin	P26040	−10
GPRC5A	G protein-coupled receptor, class C, group 5, member A	Q8BHL4	−10
POSTN	periostin, osteoblast specific factor	Q62009	−10
RPL30	ribosomal protein L30	P62889	−10
TUBB4A	tubulin, beta 4A class IVa	Q9D6F9	−10
YWHAE	tyrosine 3-monooxygenase/tryptophan 5-monooxygenase activation protein, epsilon	F6WA09	−10
GSTM1	glutathione S-transferase mu 1	P15626	−5
Macf1	microtubule-actin crosslinking factor 1	Q9QXZ0	−5
NID2	nidogen 2 (osteonidogen)	O88322	−5
PRELP	proline/arginine-rich end leucine-rich repeat protein	Q9JK53	−5
RDX	radixin	P26043	−5
RTN3	reticulon 3	Q9ES97	−5
SYPL1	synaptophysin-like 1	O09117	−5
YWHAZ	tyrosine 3-monooxygenase/tryptophan 5-monooxygenase activation protein, zeta	P63101	−5
ACADVL	acyl-CoA dehydrogenase, very long chain	P50544	−3.3
ARL8B	ADP-ribosylation factor-like 8B	Q9CQW2	−3.3
EMILIN1	elastin microfibril interfacer 1	Q99K41	−3.3
Fus	fused in sarcoma	P56959	−3.3
GNB1	guanine nucleotide binding protein (G protein), beta polypeptide 1	P62874	−3.3
SRSF1	serine/arginine-rich splicing factor 1	Q6PDM2	−3.3
SUCLG1	succinate-CoA ligase, alpha subunit	Q9WUM5	−3.3
TGM2	transglutaminase 2	P21981	−3.3
TNXB	tenascin XB	E9Q2T3	−3.3
CAV1	caveolin 1, caveolae protein, 22 kDa	P49817	−2.5
COL4A2	collagen, type IV, alpha 2	P08122	−2.5
COL6A1	collagen, type VI, alpha 1	Q04857	−2.5
EFEMP1	EGF containing fibulin-like extracellular matrix protein 1	Q8BPB5	−2.5
EIF3E	eukaryotic translation initiation factor 3, subunit E	P60229	−2.5
HP1BP3	heterochromatin protein 1, binding protein 3	Q3TEA8	−2.5
ITGA8	integrin, alpha 8	A2ARA8	−2.5
LAMB1	laminin, beta 1	P02469	−2.5
LAMB3	laminin, beta 3	Q61087	−2.5
MYL4	myosin, light chain 4, alkali; atrial, embryonic	Q9CZ19	−2.5
MYL7	myosin, light chain 7, regulatory	Q9QVP4	−2.5
SEC14L3	SEC14-like 3 (S. cerevisiae)	Q5SQ27	−2.5
AGRN	agrin	M0QWP1	−2
ATP2A2	ATPase, Ca + + transporting, cardiac muscle, slow twitch 2	J3KMM5	−2
CD36	CD36 molecule (thrombospondin receptor)	Q08857	−2
LAMA5	laminin, alpha 5	Q61001	−2
LAMB2	laminin, beta 2 (laminin S)	Q61292	−2
MYO1C	myosin IC	Q9WTI7	−2
PTRF	polymerase I and transcript release factor	O54724	−2
PC	pyruvate carboxylase	Q05920	2
RTN4	reticulon 4	Q99P72	2.2
PRDX1	peroxiredoxin 1	P35700	2.4
LCP1	lymphocyte cytosolic protein 1 (L-plastin)	Q61233	2.5
PF4	platelet factor 4	Q9Z126	2.5
DLD	dihydrolipoamide dehydrogenase	O08749	2.8
NDUFA8	NADH dehydrogenase (ubiquinone) 1 alpha subcomplex, 8, 19kDa	Q9DCJ5	2.8
GPI	glucose-6-phosphate isomerase	P06745	2.9
LTA4H	leukotriene A4 hydrolase	P24527	2.9
PSMA2	proteasome (prosome, macropain) subunit, alpha type, 2	P49722	3.2
CD93	CD93 molecule	O89103	3.3
SNRPD3	small nuclear ribonucleoprotein D3 polypeptide 18 kDa	P62320	3.4
MFAP2	microfibrillar-associated protein 2	P55002	3.9
EIF5A	eukaryotic translation initiation factor 5A	P63242	4.4
PTPRC/CD45	protein tyrosine phosphatase, receptor type, C	P06800	6.2
PGLYRP1	peptidoglycan recognition protein 1	O88593	7.6

**Table 4:** Significant changes in the lung proteome unique to *ΔpotABCD* 12 h p.i.

Protein	Description	Accession	Δ*potABCD/PBS* (Fold Change)
ITGA2	integrin, alpha 2 (CD49B, alpha 2 subunit of VLA-2 receptor)	Q62469	−5
RCN2	reticulocalbin 2, EF-hand calcium binding domain	Q8BP92	−3.3
NRP1	neuropilin 1	P97333	−3.3
ENPEP	glutamyl aminopeptidase (aminopeptidase A)	P16406	−3.3
CLDN18	claudin 18	P56857	−3.3
Cdc42	cell division cycle 42	P60766	−2.5
TUBB	tubulin, beta class I	P99024	−2
CD36	CD36 molecule (thrombospondin receptor)	Q08857	−2
PTGS1	prostaglandin-endoperoxide synthase 1 (prostaglandin G/H synthase and cyclooxygenase)	P22437	2.5
GSTP1	glutathione S-transferase pi 1	P19157	2.5
PPP2R1 A	protein phosphatase 2, regulatory subunit A, alpha	Q76MZ3	2.6
ATP6V1A	ATPase, H + transporting, lysosomal 70kDa, V1 subunit A	P50516	3.2
GUCY1B3	guanylate cyclase 1, soluble, beta 3	O54865	3.6
Irgm1	immunity-related GTPase family M member 1	Q5NCB5	3.7
FN1	fibronectin 1	P11276	3.8
TPPP3	tubulin polymerization-promoting protein family member 3	Q9CRB6	3.9
SERPING1	serpin peptidase inhibitor, clade G (C1 inhibitor), member 1	P97290	4
Igtp	interferon gamma induced GTPase	Q9DCE9	4.2
Iigp1	interferon inducible GTPase 1	Q9QZ85	6.9
HLA-DQB1	major histocompatibility complex, class II, DQ beta 1	P14483	10

**Table 5:** Relative expression changes in polyamine biosynthesis genes in *S. Pneumoniae* TIGR4 *ΔpotABCD* compared to WT TIGR4.

Gene	Description	∆*potABCD/TIGR4* (Fold Change)	p-value
*speE*	Spermidine synthase	14.4	1.81E-12
*cad*	Lysine decarboxylase	17	5.98E-12

P-values were calculated using paired two-tailed t-test.
