# Supplementary material for: Polyamine transporter in Streptococcus pneumoniae is essential for evading early innate immune responses in pneumococcal pneumonia
Source: Sci Rep. 2016 Jun 1;6:26964. doi: 10.1038/srep26964 (PMC4887915; doi:10.1038/srep26964)
Supplement: Supplementary Information [file srep26964-s1.doc]

**Polyamine transporter in *Streptococcus pneumoniae* is essential for evading early innate immune responses in pneumococcal pneumonia.**

Aswathy N. Rai^1^, Justin A. Thornton^2^, John Stokes^1^, Imran Sunesara^3^, Edwin Swiatlo^4^ and Bindu Nanduri^1*^.

^1^ Department of Basic Sciences, College of Veterinary Medicine, Mississippi State University, Mississippi State, MS, USA 39762.

^2^ Department of Biological Sciences, Mississippi State University, Mississippi State, MS, USA 39762.

^3^ Center of Biostatistics and Bioinformatics, University of Mississippi Medical Center, Jackson, MS, USA 39216.

^4^ Division of Infectious Diseases, University of Mississippi Medical Center, Jackson, MS, USA 39216.

***Corresponding author.**

**Mailing address:** College of Veterinary Medicine
P.O Box 6100
Mississippi State, MS 39762-6100.

**Phone:** 662-325-5859

**Fax:** 662-325-1031

**E-mail:** bnanduri@cvm.msstate.edu

**Figure S1:** Opsonophagocytosis assay of TIGR4 and *ΔpotABCD* at two different bacteria: neutrophil ratios (1:10 and 1:100) in the presence of Type 4 specific antibody. Murine neutrophils were incubated with Type 4 specific antibody (Hyp4M3) opsonized *S. pneumoniae*TIGR4 and *ΔpotABCD*. Viable counts relative to no neutrophil controls were used to determine percent survival. Preincubating the neutrophils with Cytochalsin-D, an actin-cytoskeleton inhibitor or heat inactivated (HI) serum had no impact on the survival of both WT and transport mutant. No serum neutrophil (100:1) control represent the reaction mixture with neutrophils and bacteria in the absence of serum and serum only control represent the reaction mixture with bacteria and serum in the absence of neutrophils. Two-Way ANOVA and Sidak’s multiple comparison test was used to calculate statistical significance (***p-value =0.0001; ****p-value = <0.0001).


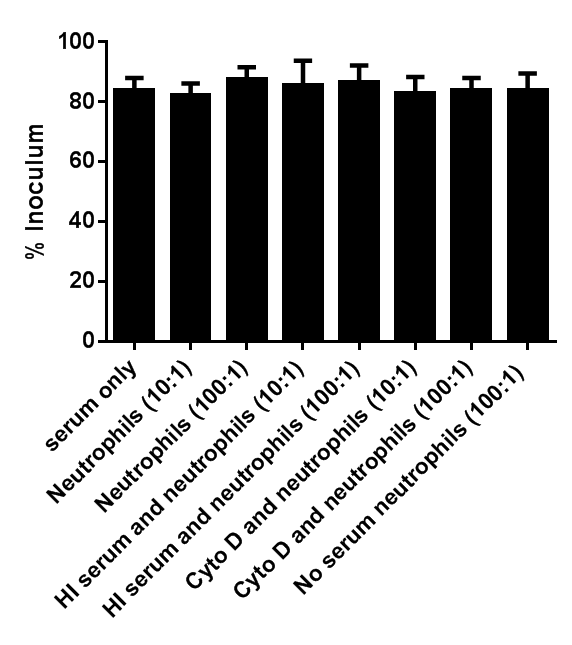

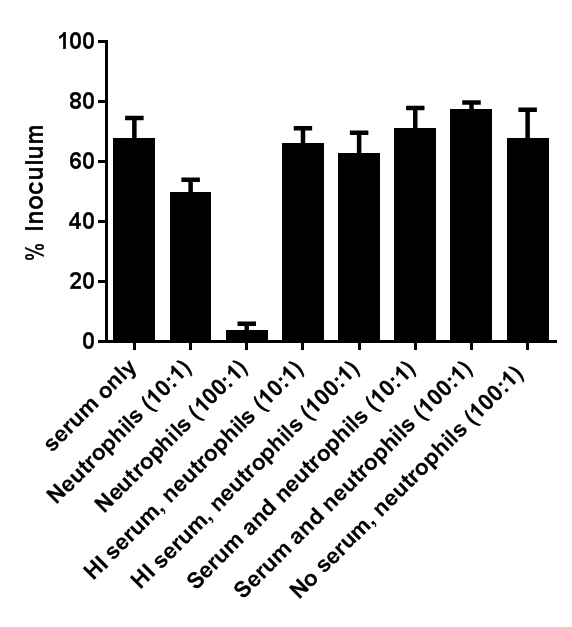
 **[A] [B]**

**Figure S2:** Opsonophagocytosis assay of TIGR4 [A] and *ΔpotABCD* [B] at two different bacteria: neutrophil ratios (1:10 and 1:100) in the absence of Type 4 specific antibody. Murine neutrophils were incubated with unopsonized *S. pneumoniae* TIGR4 [A] and *ΔpotABCD* [B]. Viable counts relative to no neutrophil controls were used to determine percent survival. Preincubating the neutrophils with Cytochalsin-D, an actin-cytoskeleton inhibitor or heat inactivated (HI) serum had no impact on the survival of both WT and transport mutant. No serum neutrophil (100:1) control represent the reaction mixture with neutrophils and bacteria in the absence of serum and serum only control represent the reaction mixture with bacteria and serum in the absence of neutrophils. Data are represented as mean ± SEM.

| ***Protein*** | ***Description*** | ***Accession*** | ***TIGR4/PBS*** |
| --- | --- | --- | --- |
| EPB41L2 | erythrocyte membrane protein band 4.1-like 2 | O70318 | -10 |
| HBB | hemoglobin, beta | E9Q223 | -10 |
| ABCD3 | ATP-binding cassette, sub-family D (ALD), member 3 | P55096 | -5 |
| FASN | fatty acid synthase | P19096 | -5 |
| ACSL1 | acyl-CoA synthetase long-chain family member 1 | P41216 | -3.3 |
| DHX9 | DEAH (Asp-Glu-Ala-His) box helicase 9 | O70133 | -3.3 |
| TNNT2 | troponin T type 2 (cardiac) | P50752 | -3.3 |
| HADHA | hydroxyacyl-CoA dehydrogenase/3-ketoacyl-CoA thiolase/enoyl-CoA hydratase (trifunctional protein), alpha subunit | Q8BMS1 | -2.5 |
| PTGIS | prostaglandin I2 (prostacyclin) synthase | O35074 | -2.5 |
| SFTPB | surfactant protein B | P50405 | -2.5 |
| TUBB2A | tubulin, beta 2A class IIa | Q7TMM9 | -2.5 |
| IMMT | inner membrane protein, mitochondrial | Q8CAQ8 | -2 |
| SERPINH1 | serpin peptidase inhibitor, clade H (heat shock protein 47), member 1, (collagen binding protein 1) | P19324 | -2 |
| FN1 | fibronectin 1 | P11276 | 2 |
| DES | desmin | P31001 | 3 |
| C4A/C4B | complement component 4B (Chido blood group) | P01029 | 3.2 |
| IFITM3 | interferon induced transmembrane protein 3 | Q9CQW9 | 3.5 |
| F2 | coagulation factor II (thrombin) | P19221 | 4.8 |
| APOE | apolipoprotein E | P08226 | 5.4 |
| LSP1 | lymphocyte-specific protein 1 | P19973 | 5.5 |
| FETUB | fetuin B | Q9QXC1 | 7.5 |
| VTN | vitronectin | P29788 | 14 |

**Table. S1** Significant changes in the lung proteome that are unique to to TIGR4 4h p.i.

| ***Protein*** | ***Description*** | ***Accession*** | ***TIGR4/***  ***PBS*** |
| --- | --- | --- | --- |
| Tmsb4x (includes others) | thymosin, beta 4, X chromosome | P20065 | -10 |
| NDUFA8 | NADH dehydrogenase (ubiquinone) 1 alpha subcomplex, 8, 19kDa | Q9DCJ5 | -10 |
| MPP1 | membrane protein, palmitoylated 1, 55kDa | P70290 | -10 |
| UQCRHL | ubiquinol-cytochrome c reductase hinge protein-like | P99028 | -5 |
| NCL | nucleolin | P09405 | -5 |
| MTHFD1 | methylenetetrahydrofolate dehydrogenase (NADP+ dependent) 1, methenyltetrahydrofolate cyclohydrolase, formyltetrahydrofolate synthetase | Q922D8 | -5 |
| Marcks | myristoylated alanine rich protein kinase C substrate | P26645 | -5 |
| LRPAP1 | low density lipoprotein receptor-related protein associated protein 1 | P55302 | -5 |
| ITGA8 | integrin, alpha 8 | A2ARA8 | -5 |
| CMKLR1 | chemerin chemokine-like receptor 1 | P97468 | -5 |
| ATP2B1 | ATPase, Ca++ transporting, plasma membrane 1 | G5E829 | -5 |
| AGK | acylglycerol kinase | Q9ESW4 | -5 |
| SLC6A14 | solute carrier family 6 (amino acid transporter), member 14 | Q9JMA9 | -3.3 |
| RCN1 | reticulocalbin 1, EF-hand calcium binding domain | Q05186 | -3.3 |
| MAOA | monoamine oxidase A | Q64133 | -3.3 |
| VDAC1 | voltage-dependent anion channel 1 | Q60932 | -2.5 |
| Rrbp1 | ribosome binding protein 1 | Q99PL5 | -2.5 |
| MAOB | monoamine oxidase B | Q8BW75 | -2.5 |
| LRRC59 | leucine rich repeat containing 59 | Q922Q8 | -2.5 |
| LGALS3 | lectin, galactoside-binding, soluble, 3 | P16110 | -2.5 |
| ITGA1 | integrin, alpha 1 | Q3V3R4 | -2.5 |
| CTNND1 | catenin (cadherin-associated protein), delta 1 | P30999 | -2.5 |
| CPT1A | carnitine palmitoyltransferase 1A (liver) | P97742 | -2.5 |
| CORO1C | coronin, actin binding protein, 1C | Q9WUM4 | -2.5 |
| CALCRL | calcitonin receptor-like | Q9R1W5 | -2.5 |
| BCAP31 | B-cell receptor-associated protein 31 | Q61335 | -2.5 |
| ITGA3 | integrin, alpha 3 (antigen CD49C, alpha 3 subunit of VLA-3 receptor) | Q62470 | -2 |
| HSD11B1 | hydroxysteroid (11-beta) dehydrogenase 1 | P50172 | 2.1 |
| RPL7 | ribosomal protein L7 | P14148 | 2.2 |
| PGAM1 | phosphoglycerate mutase 1 (brain) | Q9DBJ1 | 2.2 |
| CCT6A | chaperonin containing TCP1, subunit 6A (zeta 1) | P80317 | 2.6 |
| ALDH3A1 | aldehyde dehydrogenase 3 family, member A1 | P47739 | 2.8 |
| TTR | transthyretin | P07309 | 2.9 |
| IGHG1 | immunoglobulin heavy constant gamma 1 (G1m marker) | A0A075B5P4 | 3.5 |
| EGFR | epidermal growth factor receptor | Q01279 | 3.6 |
| KRT19 | keratin 19, type I | P19001 | 4 |
| CA3 | carbonic anhydrase III | P16015 | 4.1 |
| LCN2 | lipocalin 2 | P11672 | 5 |
| C4A/C4B | complement component 4B (Chido blood group) | P01029 | 6.2 |
| ARPC4 | actin related protein 2/3 complex, subunit 4, 20kDa | P59999 | 6.3 |
| NUCB1 | nucleobindin 1 | Q02819 | 6.4 |
| C8B | complement component 8, beta polypeptide | Q8BH35 | 7.6 |
| TTN | titin | A2ASS6 | 8.6 |
| C5 | complement component 5 | P06684 | 9.1 |
| CKMT2 | creatine kinase, mitochondrial 2 (sarcomeric) | Q6P8J7 | 11 |
| SERPINA1 | serpin peptidase inhibitor, clade A (alpha-1 antiproteinase, antitrypsin), member 1 | Q00898 | 50 |

**Table. S2:** Significant changes in the lung proteome that are unique to TIGR4 12h p.i.

| ***Protein*** | ***Description*** | ***Accession*** | ***TIGR4/PBS*** | ***ΔpotABCD/PBS*** |
| --- | --- | --- | --- | --- |
| MYH11 | myosin, heavy chain 11, smooth muscle | O08638 | -5 | -5 |
| RPS27A | ribosomal protein S27a | P62983 | -5 | -5 |
| ETFA | electron-transfer-flavoprotein, alpha polypeptide | Q99LC5 | -2.5 | -10 |
| HNRNPU | heterogeneous nuclear ribonucleoprotein U (scaffold attachment factor A) | Q8VEK3 | -2.5 | -2 |
| COL6A3 | collagen, type VI, alpha 3 | E9PWQ3 | -2 | -2.5 |
| ITGB2 | integrin, beta 2 (complement component 3 receptor 3 and 4 subunit) | P11835 | 3.4 | 4.8 |
| S100A9 | S100 calcium binding protein A9 | P31725 | 3.5 | 3.4 |
| CORO1A | coronin, actin binding protein, 1A | O89053 | 4.3 | 2.5 |
| HP | haptoglobin | Q61646 | 4.5 | 3.8 |
| CP | ceruloplasmin (ferroxidase) | Q61147 | 4.8 | 3 |
| Ces1b/Ces1c | carboxylesterase 1C | P23953 | 5.2 | 4.2 |
| S100A8 | S100 calcium binding protein A8 | P27005 | 5.2 | 4.9 |
| CYBB | cytochrome b-245, beta polypeptide | Q61093 | 5.5 | 6.9 |
| MMP9 | matrix metallopeptidase 9 | P41245 | 7.5 | 8.4 |
| Chil3/Chil4 | -- | O35744 | 11 | 9.2 |
| IGHG1 | immunoglobulin heavy constant gamma 1 (G1m marker) | A0A075B5P4 | 12 | 5.8 |
| CFH | complement factor H | P06909 | 13 | 12 |
| ELANE | elastase, neutrophil expressed | Q3UP87 | 15 | 22 |
| SERPINA1 | serpin peptidase inhibitor, clade A (alpha-1 antiproteinase, antitrypsin), member 1 | Q00897 | 16 | 1.5 |
| Ngp | neutrophilic granule protein | O08692 | 20 | 11 |
| LTF | lactotransferrin | P08071 | 22 | 26 |
| C3 | complement component 3 | P01027 | 30 | 18 |
|  |  |  |  |  |

**Table. S3:** Significant changes in the lung proteome common to TIGR4 and ∆*potABCD* at 4h p.i.

| ***Protein*** | ***Description*** | ***Accession*** | ***TIGR4/PBS*** | ***ΔpotABCD/PBS*** |
| --- | --- | --- | --- | --- |
| HLA-A | major histocompatibility complex, class I, A | P01900 | -20 | -20 |
| Nnt | nicotinamide nucleotide transhydrogenase | Q61941 | -12.5 | -11.1 |
| SNCA | synuclein, alpha (non A4 component of amyloid precursor) | O55042 | -11.1 | -10 |
| SCN7A | sodium channel, voltage gated, type VII alpha subunit | B1AYL1 | -10 | -5 |
| NDUFB4 | NADH dehydrogenase (ubiquinone) 1 beta subcomplex, 4, 15kDa | Q9CQC7 | -10 | -5 |
| NDUFA9 | NADH dehydrogenase (ubiquinone) 1 alpha subcomplex, 9, 39kDa | Q9DC69 | -10 | -3.3 |
| PHB2 | prohibitin 2 | O35129 | -5 | -10 |
| MTCH2 | mitochondrial carrier 2 | Q791V5 | -5 | -5 |
| PGRMC1 | progesterone receptor membrane component 1 | O55022 | -3.3 | -2 |
| NDUFS6 | NADH dehydrogenase (ubiquinone) Fe-S protein 6, 13kDa (NADH-coenzyme Q reductase) | P52503 | -3.3 | -2.5 |
| HNRNPL | heterogeneous nuclear ribonucleoprotein L | Q8R081 | -3.3 | -3.3 |
| EPB41 | erythrocyte membrane protein band 4.1 | P48193 | -3.3 | -2 |
| DPP4 | dipeptidyl-peptidase 4 | P28843 | -3.3 | -2.5 |
| ACE | angiotensin I converting enzyme | P09470 | -3.3 | -2.5 |
| MYOF | myoferlin | Q69ZN7 | -2.5 | -3.3 |
| HNRNPH1 | heterogeneous nuclear ribonucleoprotein H1 (H) | O35737 | -2.5 | -2 |
| CYC1 | cytochrome c-1 | Q9D0M3 | -2.5 | -2 |
| HSD17B4 | hydroxysteroid (17-beta) dehydrogenase 4 | P51660 | -2 | -2 |
| Hnrnpa1 | heterogeneous nuclear ribonucleoprotein A1 | P49312 | -2 | -2.5 |
| ALDH3A2 | aldehyde dehydrogenase 3 family, member A2 | B1AV77 | -2 | -2.5 |
| HP | haptoglobin | Q61646 | 2 | 2.8 |
| GPI | glucose-6-phosphate isomerase | P06745 | 2.2 | 2.8 |
| C3 | complement component 3 | P01027 | 2.2 | 2.8 |
| CORO1A | coronin, actin binding protein, 1A | O89053 | 2.5 | 2.4 |
| AP2A2 | adaptor-related protein complex 2, alpha 2 subunit | P17427 | 2.7 | 4.5 |
| CFH | complement factor H | P06909 | 3 | 3 |
| KRT18 | keratin 18, type I | P05784 | 3.2 | 3.7 |
| G6PD | glucose-6-phosphate dehydrogenase | Q00612 | 3.5 | 3.8 |
| LCP1 | lymphocyte cytosolic protein 1 (L-plastin) | Q61233 | 3.6 | 3.5 |
| ELANE | elastase, neutrophil expressed | Q3UP87 | 3.7 | 3 |
| CFB | complement factor B | B8JJN0 | 4 | 3.5 |
| S100A9 | S100 calcium binding protein A9 | P31725 | 4.2 | 3.2 |
| LTF | lactotransferrin | P08071 | 4.5 | 3.5 |
| PGLYRP1 | peptidoglycan recognition protein 1 | O88593 | 4.8 | 3.8 |
| ITGA2B | integrin, alpha 2b (platelet glycoprotein IIb of IIb/IIIa complex, antigen CD41) | Q9QUM0 | 4.8 | 6.1 |
| ITIH4 | inter-alpha-trypsin inhibitor heavy chain family, member 4 | A6X935 | 5.5 | 8.7 |
| FETUB | fetuin B | Q9QXC1 | 5.5 | 3.1 |
| S100A8 | S100 calcium binding protein A8 | P27005 | 5.9 | 4.9 |
| CP | ceruloplasmin (ferroxidase) | Q61147 | 6.5 | 4.4 |
| CYBB | cytochrome b-245, beta polypeptide | Q61093 | 6.8 | 4.1 |
| ARHGDIB | Rho GDP dissociation inhibitor (GDI) beta | Q61599 | 6.8 | 7 |
| SERPINB1 | serpin peptidase inhibitor, clade B (ovalbumin), member 1 | Q9D154 | 9 | 8.7 |
| ITGB2 | integrin, beta 2 (complement component 3 receptor 3 and 4 subunit) | P11835 | 9.6 | 8 |
| DES | desmin | P31001 | 12 | 10 |
| Ngp | neutrophilic granule protein | O08692 | 18 | 16 |
| Chil3/Chil4 | -- | O35744 | 37 | 27 |

**Table. S4:** Significant changes in the lung proteome common to TIGR4 and ∆*potABCD* at 12h p.i.

| ***Primer*** | ***Sequence*** |
| --- | --- |
| ***Gdh (For)*** | CAACTGATGAAGAACTCAAAGAACAC |
| ***Gdh (Rev)*** | TGGCTCGCTACGATAAGAGATG |
| ***speE (For)*** | TGCGGATGATTTCGTCTACAATG |
| ***speE (Rev)*** | CCAGTTCAGGATAGAGGGTTAATAC |
| ***cadA (For)*** | AGTCGTTTGAGAAAGTCATTGAGC |
| ***cadA (Rev)*** | AAACCGAAACACCGTCTATTAACTC |

**Table S9.** List of primers and their sequence used for *in vitro* analysis of *speE* and *cadA* genes.

**Supplementary methods**

**Proteomics**

**Protein isolation and sample preparation for mass spectrometry**

Lungs were harvested from infected and sham treated (PBS) animals (n=3) 4h, 12h p.i. Lung tissue was homogenized in PBS on ice. Lung homogenates were clarified by centrifugation at 2500g for 15 min at 4° C and pellets were stored at -80° C. Total proteins were isolated from triplicate tissue pellets from uninfected, TIGR4, and *ΔpotABCD* infected groups by incubation in NP-40 lysis buffer (0.5% NP-40, 150 mM NaCl, 20 mM CaCl2·2H_2_O, 50 mM Tris, pH 7.4) supplemented with 1 mM serine protease inhibitor phenylmethylsulfonyl fluoride, for 20 min on ice. The tissue debris was removed by centrifugation at 17000g, at 4°C for 10 min. Protein concentration from the supernatant was determined using Pierce BCA Protein Assay Kit. Samples containing 100 µg protein were precipitated with methanol and chloroform (4:1), washed with methanol, spun and vacuum dried.  Protein samples were solubilized in 8M urea, reduced (0.005 M DTT at 65° C for 10 min) and alkylated (0.01 M iodoacetamide at 37° C for 30 min). Protein samples were diluted with water, pH was adjusted to 7.5 and they were digested with molecular biology grade porcine trypsin (2 µg at 37°C, overnight, 50:1 ratio of protein:trypsin, Promega Corporation, Madison, WI).  Tryptic peptides were acidified with trifluoroacetic acid and desalted using a C8 peptide macrotrap (Michrom BioResources Inc., Auburn, CA) with 70% formic acid, 30% isopropanol (for regeneration) and 2% acetonitrile, 0.1% trifluoroacetic acid (for equilibration), eluted in 0.1% triflouroacetic acid, 95% acetonitrile and vacuum dried.

**Mass spectrometry**

The desalted tryptic peptides were resuspended in 0.1% formic acid and ~500 ng was subjected to 1D nano-LC ESI MS/MS analysis using an iontrap LTQ mass spectrometer. Liquid chromatographic (LC) analysis to separate peptides was performed using a 75-µm i.d. x 10 cm reverse phase C18 column (Thermo Scientific, Waltham, MA) controlled by an Proxeon Easy n-LC (Thermo Scientific, Waltham, MA). Peptides were eluted using a 148 min gradient from 5%-40% solvent B (99.9% acetonitrile, 0.1% formic acid) followed by a 5 min ramp to 85% for 10 min. The gradient then returned to 5% B for 1 min and held at this percentage of solvent B for 16 min for a total gradient time of 180 min at a flow rate of 400nl/min. LC flow is directed to Advion TriVersa Nanomate nanospray source (Advion, Ithaca, NY) at 1.75 kV and introduced into an LTQ-OrbiTrap Velos mass spectrometer (Thermo Scientific, Waltham, MA). The mass spectrometer was set to acquire data for the entire 180 min of the LC gradient. Full scan MS spectra (60,000 resolution and m/Z 350-1,600 amu) were analyzed by Orbitrap, and the ions were selected for collision-induced fragmentation (CID) in the LTQ at normalized collision energy of 35% and activation time of 30 msec. Data dependent settings include enabling FT master scan preview, rejection of unassigned charge states and charge state 1 and selection of monoisotopic precursor. Dynamic exclusion settings allowed one MS/MS acquisition followed by placing the m/z on an exclusion list for 45 sec at exclusion mass width of 10 ppm low and high.

**Data analysis**

Tandem mass spectra were extracted, charge state deconvoluted and deisotoped by Thermo Proteome Discoverer 1.3 (Thermo Scientific, Waltham, MA). Sequest was set up to match mass spectra and tandem mass spectra against a non-redundant protein database (47,393 proteins) that had mouse proteins appended with *S. pneumoniae* proteins (both downloaded from Uniprot) and common laboratory contaminants downloaded from the global proteome machine (<http://www.thegpm.org/cRAP/>). A decoy database that had all these protein sequences reversed was also utilized to assess false discovery rate for peptide identification. Sequest searches were set up with precursor masses 500-5000 Da, with fully tryptic peaks with a mass tolerance of 10 ppm for precursor masses and 0.8 Da for fragment ions. Two missed cleavages were allowed for trypsin. Dynamic modifications considered were carbamidomethylation of cysteine (57.021 Da) and oxidation of methioinine (15.995 Da). Individual peptide spectra were scored using Sequest cross-correlation (XCorr) scores, and False Discovery Rate scores were determined from the decoy search at 0.01 (strict) and 0.05 (relaxed). Discoverer results were then loaded to Scaffold 4 (Proteome Software, Portland, OR) and filtered for peptide and protein identifications and differential expression analysis. Peptide identifications were accepted if they could be established at greater than 92.0% probability to achieve an FDR less than 0.5% by the Scaffold Local FDR algorithm (see supplementary tables S5B-8B). Protein identifications were accepted if they could be established at greater than 99.0% probability and contained at least 2 identified peptides (see supplementary tables S5A-8A). Protein probabilities were assigned by the Protein Prophet algorithm^1^. Proteins that contained similar peptides and could not be differentiated based on MS/MS analysis alone were grouped to satisfy the principles of parsimony. Proteins sharing significant peptide evidence were grouped into clusters. Significant changes in protein expression between uninfected control versus TIGR4 and uninfected control versus *ΔpotABCD* at 4h and 12h were identified by Fisher’s exact test at a p-value of ≤ 0.05. Fold changes in protein expression between the comparison groups were calculated using weighted normalized spectra and 0.5 imputation value and a fold change cut off of 1.5 was used to generate a list of proteins for biological interpretation. The PRoteomics IDEntifications (PRIDE) database is a centralized, standards compliant, public data repository for proteomics data. The mass spectrometry proteomics data have been deposited to the ProteomeXchange Consortium ^2^ via the PRIDE partner repository with the dataset identifier PXD002300 and 10.6019/PXD002300.

**Ingenuity pathways analysis**

In order to identify the molecular functions, signaling pathways, and networks represented by significantly altered host proteins during infection with TIGR4 and *∆potABCD*, we analyzed differentially expressed proteins using Ingenuity Pathways Analysis (IPA, Qiagen, Valencia, CA ) as was done earlier ^3^. Briefly, IPA mapped proteins from our datasets to gene objects (focus genes) in the Ingenuity Pathways Knowledgebase (IPKB) known to interact with other genes in networks and pathways. Fisher exact test is used to calculate the P-value determining the probability of each biological function/disease or pathway being assigned by chance. We used P ≤ 0.05 to select highly significant biological functions and pathways represented in our proteomic datasets. IPA computes a score for each network from P-value and indicates the likelihood of the focus genes in a network being found together due to chance. We selected networks scoring ≥ 2, which have > 99% confidence of not being generated by chance. IPA predicts activation of upstream and downstream regulators to help determine which causal relationships previously reported in the literature could explain the observed changes in protein expression and provides a Z-score for this predicted activation/inhibition ^4^. A Z-score of ≥ +/- 2 was considered significant. The regulator effects algorithm connects such identified upstream regulators with proteins in a dataset and downstream functions to generate a hypothesis and ranks several regulator effects hypotheses by consistency score, which indicates how causally consistent and densely connected a regulator effects network is. A higher consistency score indicated consistency in the predicted functions based on published literature.

**Supplementary References**

1 Nesvizhskii, A. I., Keller, A., Kolker, E. & Aebersold, R. A statistical model for identifying proteins by tandem mass spectrometry. *Anal Chem* **75**, 4646-4658 (2003).

2 Jones, P. *et al.* PRIDE: a public repository of protein and peptide identifications for the proteomics community. *Nucleic acids research* **34**, D659-663 (2006).

3 Peddinti, D. *et al.* Comprehensive proteomic analysis of bovine spermatozoa of varying fertility rates and identification of biomarkers associated with fertility. *BMC systems biology* **2**, 19, doi:10.1186/1752-0509-2-19 (2008).

4 Kramer, A., Green, J., Pollard, J., Jr. & Tugendreich, S. Causal analysis approaches in Ingenuity Pathway Analysis. *Bioinformatics* **30**, 523-530, doi:10.1093/bioinformatics/btt703 (2014).
